# Induction of anti-aging gene klotho with a small chemical compound that demethylates CpG islands

**DOI:** 10.18632/oncotarget.18608

**Published:** 2017-06-22

**Authors:** Dongju Jung, Yuechi Xu, Zhongjie Sun

**Affiliations:** ^1^ Department of Physiology, College of Medicine, University of Oklahoma Health Sciences Center, Oklahoma City, OK, USA; ^2^ Current address: Department of Biomedical Laboratory Science, Hoseo University, Chungnam, Republic of Korea

**Keywords:** klotho, methylation, Pax4, Kid3, CpG island

## Abstract

Klotho (KL) is described as an anti-aging gene because mutation of *Kl* gene leads to multiple pre-mature aging phenotypes and shortens lifespan in mice. Growing evidence suggests that an increase in KL expression may be beneficial for age-related diseases such as arteriosclerosis and diabetes. It remains largely unknown, however, how *Kl* expression could be induced. Here we discovered novel molecular mechanism for induction of *Kl* expression with a small molecule ‘Compound H’, *N*-(2-chlorophenyl)-1*H*-indole-3-caboxamide. Compound H was originally identified through a high-throughput screening of small molecules for identifying *Kl* inducers. However, how Compound H induces *Kl* expression has never been investigated. We found that Compound H increased *Kl* expression *via* demethylation in CpG islands of the *Kl* gene. The demethylation was accomplished by activating demethylases rather than inhibiting methylases. Due to demethylation, Compound H enhanced binding of transcription factors, Pax4 and Kid3, to the promoter of the *Kl* gene. Pax4 and Kid3 regulated *Kl* promoter activity positively and negatively, respectively. Thus, our results show that demethylation is an important molecular mechanism that mediates Compound H-induced *Kl* expression. Further investigation is warranted to determine whether Compound H demethylates the *Kl* gene *in vivo* and whether it can serve as a therapeutic agent for repressing or delaying the onset of age-related diseases.

## INTRODUCTION

Pre-mature aging phenotypes were eminent in the *klotho (Kl)*-deficient mice, which have ~ 10 copies of a transgene integrated in the 5’ flanking region of the *Kl* gene disrupting its expression [1]. The *klotho* mice die around ~ 2 months of age after birth due to multiple aging-related organ failures [1]. Later, the role of KL in aging was confirmed by the reproduction of the same aging phenotypes in *Kl* knockout homozygous (Kl −/−) mice [2]. On the other hand, overexpression of KL extends lifespan by 20-30% [2, 3]. The protein products of *Kl* gene can be divided into two forms: membrane-integrated form of Kl and non-integrated form of Kl which includes secreted and soluble Kl (sKl). These two type of proteins are produced from the two transcripts that arise from a single *kl* gene due to alternative RNA splicing [4, 5]. It has been reported that sKl also can be produced by enzymatic cleavage of the extracellular domain of membrane Kl [5–7]. The membrane-integrated Kl is a single-pass transmembrane protein composed of a short intracellular domain and a long extracellular domain that resembles β-glucuronidase [8]. The membrane Kl functions as a co-receptor for fibroblast growth factor-23 (FGF23), while sKl is believed to have diverse systemic roles and function as a hormone [5, 9–11].

Kl is primarily expressed in brain and kidney, particularly at choroid plexus and distal tubules, respectively [5, 11]. Two other *Kl*-related genes have been identified based on the DNA sequence similarity; they are designated as β- and γ-klotho, respectively [12]. They are type I transmembrane proteins and behave as coreceptors of FGFs similar to the original Kl which is designated as αKl, but they are expressed in different tissues [13].

The KL expression level decreases proportionally with aging, but molecular mechanisms for the decrease are largely unknown [1, 3]. An increase of DNA methylation on the *Kl* CpG islands was found in aged rhesus monkeys [14]. Overexpression of *Kl* was able to rescue the aging phenotypes in *Kl*-deficient mice. Therefore, induction of *Kl* might be an effective therapeutic strategy for the onset of aging-related diseases. Several transcription factors and small molecules were reported as *Kl* inducers. Vitamin D receptor (VDR), Egr1 (early growth response protein 1), and peroxisome proliferator activation receptor γ (PPARγ) are the reported transcription factors for klotho transcription [15–17]. Klotho expression can be induced by rapamycin, 25-OH vitamin D, and statins - inhibitors for HMG-CoA reductase [17–20]. Because of the powerful anti-aging effect of klotho, it will be intriguing to explore the molecular mechanism of induction of klotho.

In an effort to find molecular mechanisms for *Kl* induction, we examined the small molecules and transcription factors that bind to the promoter/enhancer sequences of *Kl* gene. Candidate transcription factors were selected by analyses of the 2.0 kb upstream DNA sequences of human and mouse *Kl* genes using TRANSFAC^®^. Transactivation functions of the selected transcription factors and small molecules were investigated using a reporter plasmid in which firefly luciferase gene expression was under control of human *KL* promoter/enhancer [21]. We found an activation mechanism for induction of *Kl* expression by a small chemical compound H. The induction by Compound H was accomplished through demethylation on the CpG islands of *Kl* gene and subsequent binding of transcription factors to the promoter. These results suggest that artificial DNA demethylation could be an activation mechanism for *Kl* expression.

## RESULTS

### Expression levels of the candidate transcription factors

**Figure 1:**
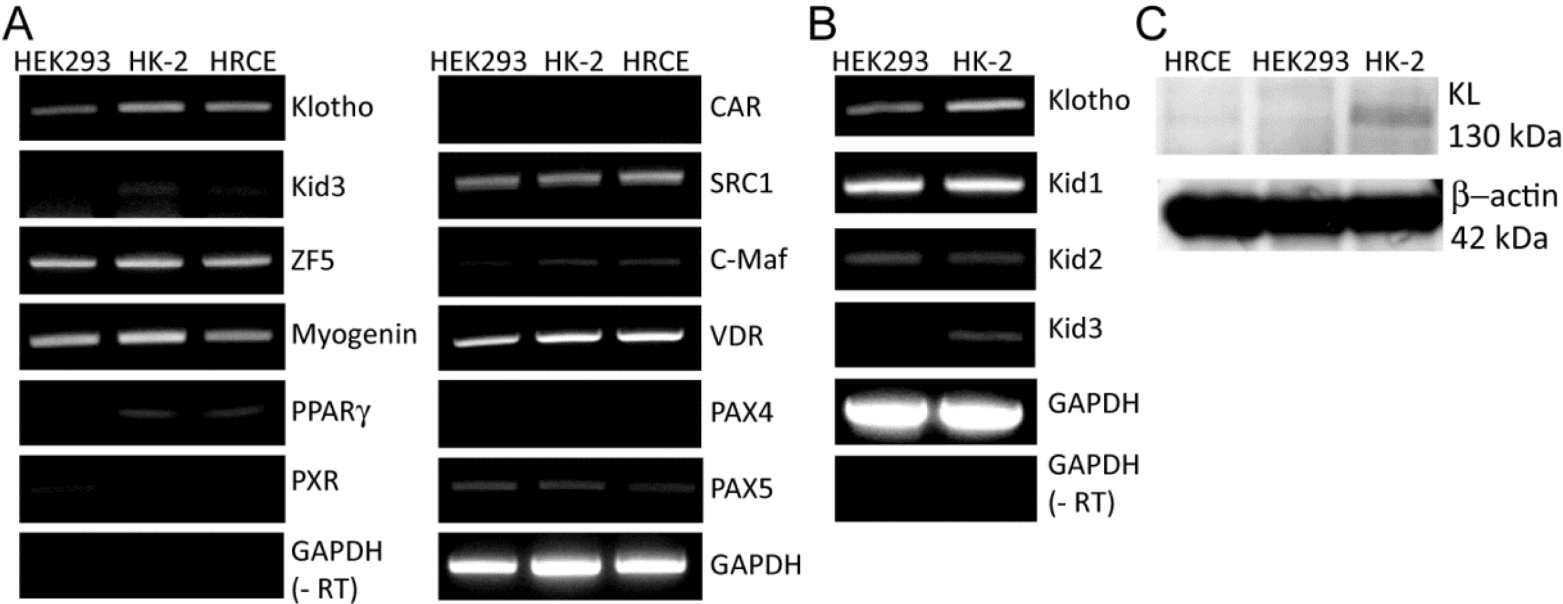
Expression levels of the candidate transcription factors (TFs) **A**. Expression levels of the identified candidate TFs were analyzed by semi-quantitative reverse transcription-polymerase chain reaction (RT-PCR). Total RNA (1 μg) purified from the kidney cell lines, HEK293, HK-2, and HRCE, were used to synthesize the first cDNA, which was used as a template DNA for the PCR reactions. **B**. Expression levels of the Kid family TFs and klotho (KL) in HK-2 cells were compared with their expression levels in HEK293 cells by semi-quantitative RT-PCR. (-RT) indicates a negative control that was processed equally with the other experiment except reverse transcriptase. **C**. Whole cell lysates (10 μg) from the three cell lines were used for quantifying endogenous expression level of KL protein.

Human and mouse *KL* gene promoter/enhancer sequences up to -2.0 kb from the first ATG codon were analyzed with TRANSFAC^®^ from Biobase to deduce candidate transcription factors (TFs) that could occupy the promoter sequences. Under the highest stringency, total 36 and 35 putative transcription factors were obtained through the analyses of human and mouse klotho gene promoter/enhancer region, respectively. TFs commonly identified for human and mice were selected for further analyses, which includes Kid3 (kidney transcription factor-3), Pax4 (paired box gene 4), ZF5 (zinc finger transcription factor-5), and CTCF (CCCTC-Binding factor) (Table 1). Expression levels of these TFs and conventional coactivators were analyzed using kidney cell lines such as HEK293 (human embryonic kidney cells), HRCE (human cortical cells that are mixture of proximal tubule and distal tubule), and HK-2 (epithelial cells from human kidney proximal tubule). All of the selected TFs except kid3 were expressed equally in the three kidney cell lines (Figure 1A). The expression level of kid3 was lower in HEK293 cells than that in other cell lines (Figure 1A). There are three kid TFs in the kid family, therefore we investigated expression levels of other kid TFs, kid1 and kid2. Among the kid TFs, kid3 was the only TF expressed lower in HEK293 cells (Figure 1B). Expression levels of KL mRNA and protein were higher in HK-2 cells than in other types of cells (Figure 1A, 1C). Consistent with the mRNA level, the protein level of Kid3 (ZFP354C) was lower in HEK-293 cells than in HK-2 cells, whereas there was no significant difference in the expression level of ZF5 (ZFP161) protein between the cell lines (Supplementary Figure 1A & B).

**Table 1:** Candidate transcription factors and their response elements in the KL promoter region

Transcription factor	Response element
Kid3	CGTGG or GGTGG
Pax4	gGGGTGggcacc
ZF5	ggggcgCGGGCat
CTCF	CCCTC

### Screening of the small molecules

**Figure 2:**
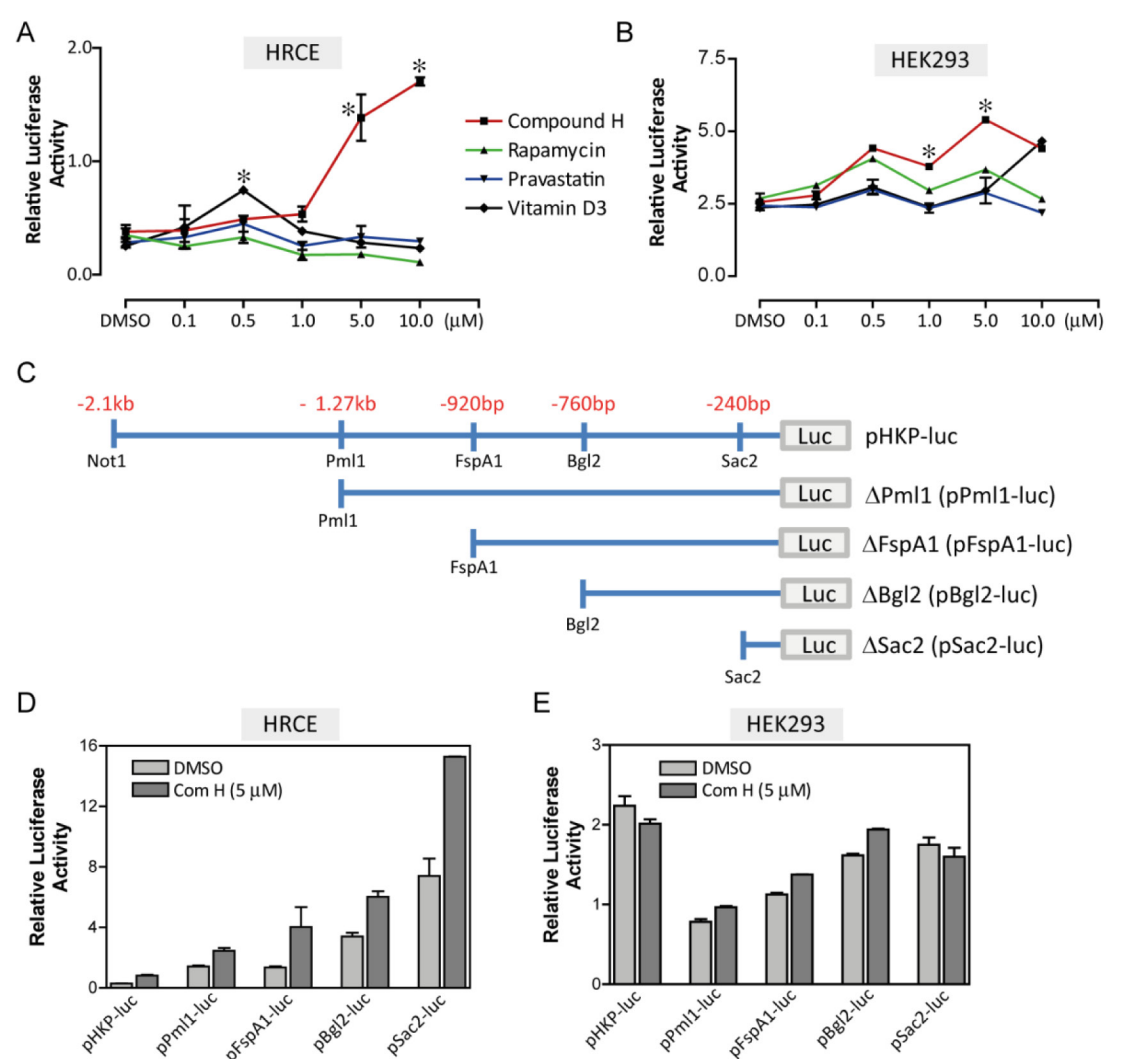
Screening of the small molecules HRCE cells or HEK293 cells were transiently transfected with two reporter plasmids, one contained a *KL* promoter and the other contained thymidine kinase (tk) promoter as a control. Dose-response curves for the small molecules in HRCE cells **A**. and HEK293 cells **B**.. **C**. Fragmented KL promoter used for constructing the reporter plasmids. Effects of Compound H on luciferase activity of fragmented reporter plasmids were examined in HRCE cells **D**. and HEK293 cells **E**.. *indicates statistically significant (*p* < 0.01) induction of the reporter expression by the small molecules. *N* = 3 independent experiments.

The selected TFs were functionally tested using a reporter plasmid (pHKP-luc) in which fire fly luciferase gene was expressed under the control of human *KL* promoter [21]. Marginal repression and activation of the reporter expression were induced by overexpression of kid3 and ZF5, respectively (Supplementary Figure 2). We believed that simple overexpression of a TF might not be strong enough to induce endogenous *KL* expression. Therefore, we focused on the small molecules that were reported to induce *KL* expression using the same reporter plasmids. Rapamycin, pravastatin, vitamin D3, and compound H were selected according to the previous reports [18–20, 22]. These small molecules were not able to induce the reporter expression strongly except compound H. Compound H increased expression of the pHKP-luc reporter by 7-folds in HRCE cells (Figure 2A). Interestingly, this strong activation effect of compound H was not observed in HEK293 cells; compound H induced only 2-fold induction of the reporter expression in HEK293 cells with an irregular dose-response curve (Figure 2B). To define a response element for compound H, the pHKP-luc reporter plasmid was modified to have a shorter fragment of the promoter sequence (Figure 2C). Through transient transfection assays, it was confirmed that compound H treatment induced expression of all the reporter plasmids even one having the shortest response element, pSac2-luc, in HRCE cells (Figure 2D), but failed to induce obvious expression of any reporter plasmids in HEK293 cells (Figure 2E). Thus, the effect of compound H on the *KL* promoter activation varies with cell lines.

### Induction of endogenous KL expression by compound H

**Figure 3:**
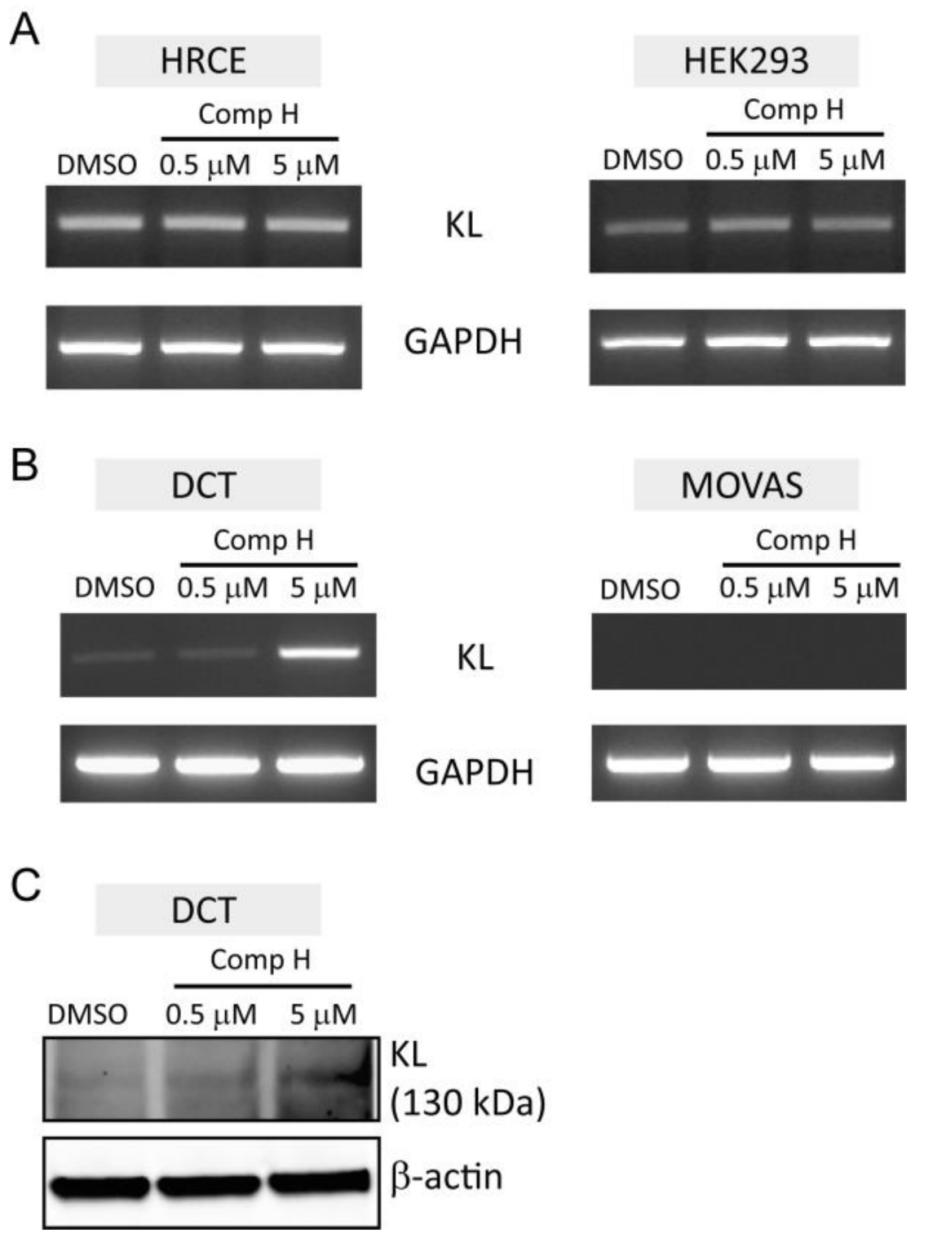
Induction of endogenous *KL* expression by compound H mRNA purified from the cells treated with DMSO or compound H was used for the semi-quantitative PCR. **A**. KL and GAPDH expression levels in the human cell lines, HRCE cells and HEK293 cells, following treatment with compound H for 6 hr. **B**. Expression levels of *Kl* and *GAPDH* in the mouse cell lines, DCT and MOVAS, following treatment with compound H for 6 hr. **C**. Expression levels of Kl protein in DCT cells treated with compound H were measured by western blot.

Next we examined whether compound H could induce endogenous *KL* expression. Total RNA was purified from mammalian cells treated with DMSO as a control or compound H for analyzing *KL* mRNA expression. It was uncovered through semi-quantitative PCR experiments that endogenous *KL* expression was not induced by compound H treatment in the human cell lines originated from kidney, such as HRCE and HEK293 cells (Figure 3A). A mouse cell line originated from distal convoluted tubule (DCT) in the kidney increased expression of *Kl* mRNA in response to compound H treatment (Figure 3B). The *Kl* mRNA expression was absent in a mouse vascular smooth muscle cell line (MOVAS), indicating that *Kl* is not expressed in smooth muscles (Figure 3B). Compound H did not affect Kl expression (Figure 3B). The Kl protein expression was also increased in the DCT cells treated with compound H in accordance with the *Kl* mRNA induction (Figure 3C). These results suggest that endogenous *Kl* expression in mammalian cells can be induced by compound H. However, the *Kl* induction was limited to the distal convoluted cells.

### Compound H increased binding of Kid3 and Pax4 to the KL promoter

**Figure 4:**
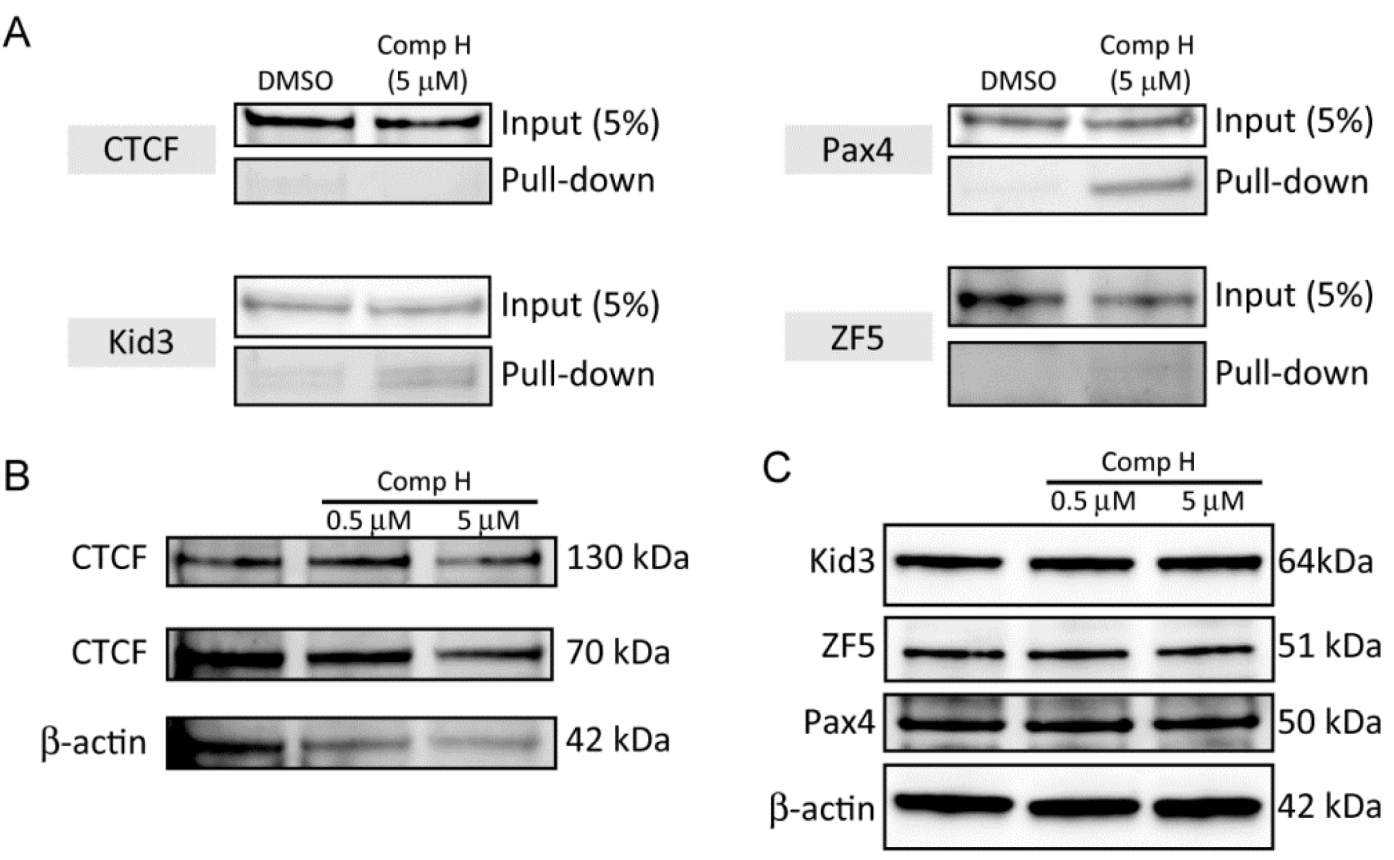
Compound H increased binding of Kid3 and Pax4 to the *KL* promoter **A**. Nuclear extracts (1 mg) of DCT cells treated with DMSO or compound H (5 μM) for 24 hr were incubated with the 200 bp DNA containing the *KL* promoter fragment. And the binding proteins were subject to analyses of binding of the TFs to the DNA by western blot **B**.. Whole cell extracts (10 μg) from the DCT cells treated with DMSO or compound H for 24 hr were loaded onto each well for western blot analyses of protein expression levels of transcription factors. The 70 kDa CTCF is the major fragment of full-length (130 kDa) CTCF.

We investigated whether the candidate TFs are involved in compound H-induced signaling to activate *KL* expression. Specifically, we examined whether binding of the TFs toward *KL* promoter could be changed by compound H. To this end, we employed a DNA pull-down assay. Magnetic beads coated with streptavidin protein were labeled with the biotin-conjugated DNA fragment encompassing 200 bp upstream from the first ATG codon of human *KL* gene. The 200 bp DNA contains all the response elements for the TFs. The beads were incubated with nuclear extracts purified from DCT cells treated with DMSO or 5 μM compound H. Binding proteins were recovered using a magnet and were subject to analysis with SDS-PAGE and western blot. The binding of the two TFs, Kid3 and Pax4, to the 200 bp DNA was increased by compound H, whereas binding affinity of CTCF and ZF was not increased (Figure 4A). The total cellular protein expression levels of the TFs, such as ZF5, Pax4, Kid3 and CTCF, were not changed by compound H treatment (Figure 4B).

These results indicate that the *KL* induction mechanism of compound H may not be through the increase of the TFs, but through the increased binding of these selected TFs (Kid3, Pax4) to the *KL* promoter.

### Two transcription factors, Pax4 and Kid3, mediated the effects of compound H

**Figure 5:**
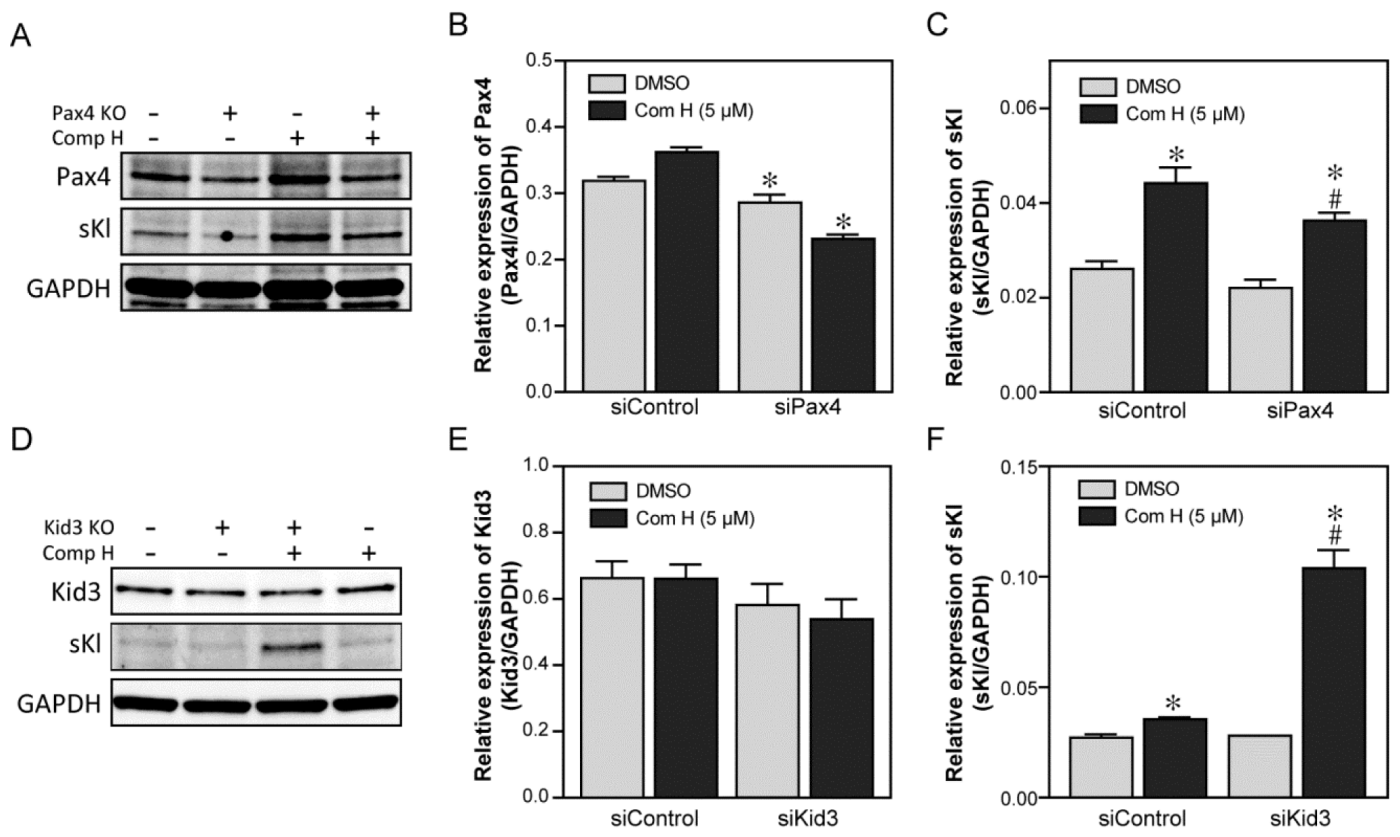
Two transcription factors, Pax4 and Kid3, mediated the effects of compound H Effects of the two transcription factors, Pax4 and Kid3, on the compound H-induced induction of Kl were analyzed following siRNA-mediated suppression. DCT cells were transfected with siRNA for Pax4 or Kid3, and then treated with DMSO or compound H. Whole cell extracts were collected for protein expression analyses with specific antibodies. **A**. Representative western blot results for Pax4 and secreted Kl (sKl) expression in the Pax4-supressed DCT cells. **B**. Quantification of Pax4 expression. *indicates statistically significant ( *p* < 0.05) suppression of Pax4 by siPax4 transfection (*vs*. siControl). **C**. Quantification of the sKl expression. *indicates statistically significant (*p* < 0.01) induction of sKl by compound H treatment (*vs*. DMSO). # indicates statistically significant (*p* < 0.01) decrease of the compound H-induced sKl expression in siPax4-transfected cells compared with that in control siRNA-transfected cells treated with compound H. **D**. Representative western blot results of Kid3 and sKl expression in Kid3-suppressed DCT cells. **E**. Quantification of the Kid3 expression. **F**. Quantification of sKl expression. *indicates statistically significant ( *p* < 0.05) induction of sKl by compound H treatment.# indicates a statistically significant (*p* < 0.01) increase of the compound H-induced sKl expression in the siKid3-transfected cells compared with that in the control siRNA-transfected cells treated with compound H. *N* = 3 independent experiments.

Because binding of Kid3 and Pax4 to the *KL* promoter was increased by compound H treatment, we next investigated whether Kid3 or Pax4 are functionally involved in compound H-induced activation of Kl expression. To this end, DCT cells were transfected with siRNA for Kid3 or Pax4 to suppress expression of these transcription factors. A scrambled sequence of siRNA was used as a control siRNA. The Pax4 expression level was suppressed in the siPax4-transfected cells (Figure 5A, 5B) which attenuated the induction of secreted Kl (sKl) by compound H (Figure 5A, 5C). Kid3 suppression by siKid3 was hard to detect (Figure 5D, 5E): many transfected cells were floated and dead (data not shown). In a low concentrated transfection of the Kid3 siRNA, we were able to detect that Kid3 suppression significantly potentiated compound H-induced induction of *sKl* expression (Figure 5D, 5F). These results suggest that Pax4 and Kid3 are involved in induction of Kl expression positively and negatively, respectively, by compound H.

### Effect of compound H on DNA demethylation

**Figure 6:**
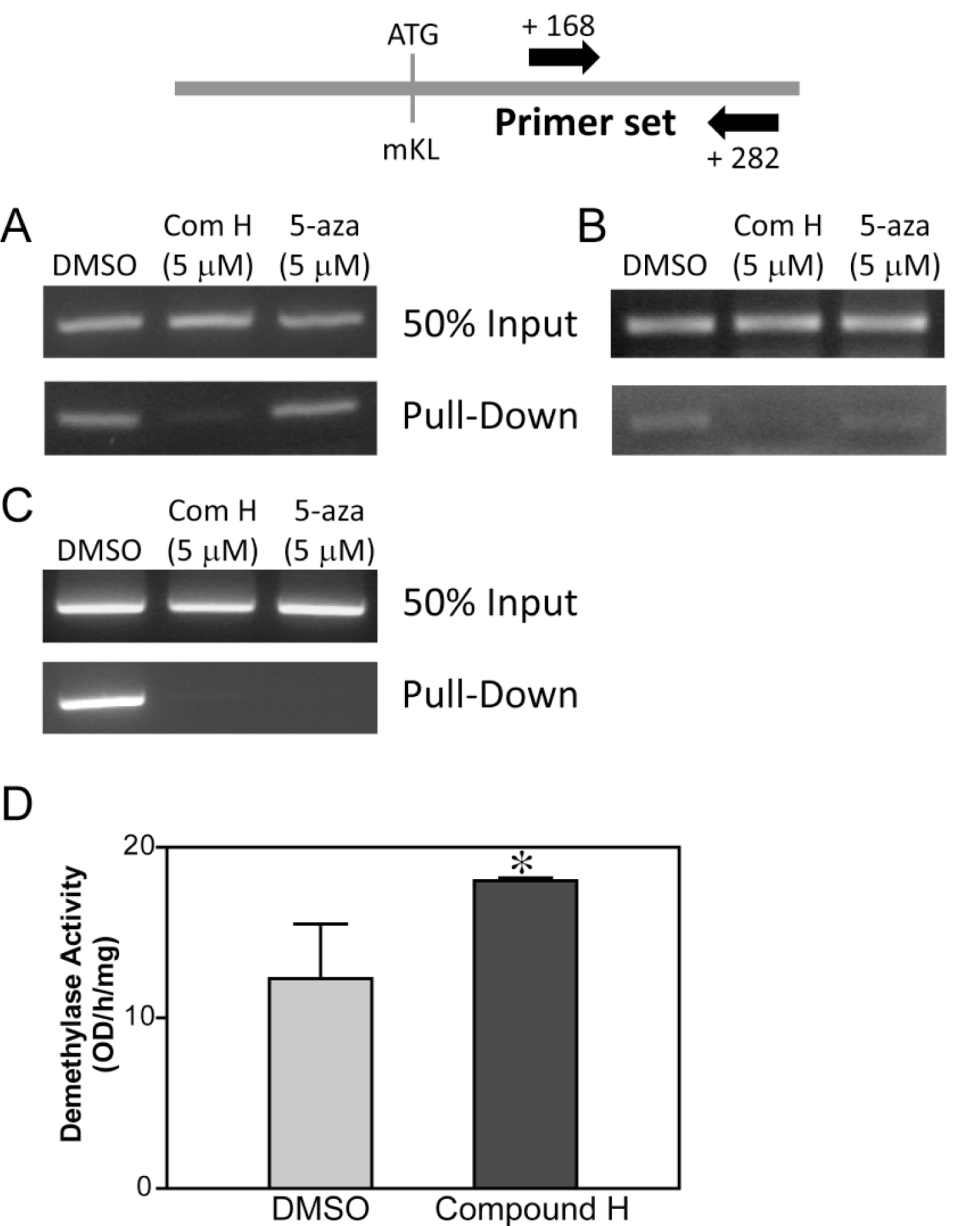
Effect of compound H on DNA demethylation Genomic DNA was purified from DCT cells treated with compound H. After fragmentation with Mse 1, the genomic DNAs were incubated with Mecp2 protein to capture the methylated DNA fragments, which were used to amplify CpG islands on *Kl* or *p16*. PCR amplification of the CpG islands on the mouse *Kl* gene using purified methylated DNA from DCT cells were treated with compound H (Com H) or 5-azacytidine (5-aza) for 24 hr **A**. or 6 hr **B**.. **C**. CpG island on the *p16* gene was amplified by PCR as described above. **D**. The demethylase enzyme activity was measured using nuclear extracts from DCT cells treated with DMSO or compound H for 24 hr. *n* = 3. *indicates statistically significant (*p* < 0.01) change of demethylase enzyme activity in the DCT cells treated with compound H (*vs*. DMSO).

It has been known that the *Kl* expression level decreases with aging probably due to an increase of DNA methylation on the CpG islands of *Kl*. Therefore, demethylation could be a mechanism for induction of *Kl* expression. We examined the methylation levels on the CpG islands of *Kl* following treatment with compound H. The DNA methylation status was quantitatively measured using a kit, with which methylated DNA was selectively captured by Mecp2, a protein exclusively binds to methylated DNA, and an anti-Mecp2 antibody-labeled beads. Eluted methylated DNA was used to amplify CpG islands of *Kl* with specific primers for the known methylated CpG region. For this experiment, we designed a primer set for amplifying between +168 and +282 from the first ATG codon of mouse *Kl*. Methylation on the CpG island of *Kl* was decreased in the DCT cells treated with compound H for 24 hr, whereas treatment with a DNA methylase inhibitor ‘5-azacytidine’ failed to decrease methylation (Figure 6A). The demethylation effect of compound H on *Kl* was detected in DCT cells as early as 6 hours after treatment with compound H (Figure 6B). The methylation status on p16 (*CDKN2A*) gene was measured to determine whether the demethylation effect of compound H was exclusive to *Kl*. Similar to its effect on *Kl*, compound H treatment induced demethylation of CpG islands of p16 gene (Figure 6C). Methylation of p16 gene was also decreased by 5-azacytidine treatment which was not observed for the *Kl* gene (Figure 6A and 6C).

We expected that compound H might be an activator for DNA demethylases rather than a methylase inhibitor such as 5-azacytidine because compound H was able to induce the demethylation in such a short period of the incubation time (6hr). To confirm it, enzyme activity of DNA demethylases was measured using nuclear extracts purified from the DCT cells treated with compound H for 6 hr. DNA demethylase activity was measured by quantifying left over methylated DNA amounts with an anti-methyl cytosine antibody after incubation with the nuclear extracts. The DNA demethylase activity in the DCT cells treated with compound H was increased significantly in DCT cells (Figure 6D).

These results indicate that compound H induced *Kl* expression through demethylation on CpG islands of the *Kl* gene by enhancing the demethylase activity.

## DISCUSSION

Klotho is an aging-suppressor gene [5]. Emerging evidence suggests that induction of klotho expression may be an effective therapeutic strategy for preventing or ameliorating aging-associated diseases [3, 23–27]. However, it remains a challenge to induce KL expression for the therapeutic purposes owing to insufficient knowledge of the induction mechanism. This study demonstrates a new molecular mechanism for induction of *Kl* expression, i.e., demethylation of *Kl* gene, by a small molecule called compound H. Compound H was reported to induce *Kl* expression but its induction mechanism has never been explored [20]. We found that compound H activated the expression of a reporter plasmid, which was composed of human *KL* gene promoter followed by luciferase gene [21]. The effect of compound H in induction of KL expression was much stronger than that of 1,25-dihydroxy vitamin D (calcitriol), an agonist ligand for vitamin D receptors and is known to induce *KL* expression [17, 19]. Calcitriol induces KL expression through vitamin D receptor response element existing in the *KL* promoter. Therefore, it is highly likely that calcitriol may be an endogenous inducer of *KL* expression because calcitriol expresses in the proximal tubule in the kidney that is physically close to the distal tubule where *KL* is expressed. We found that calcitriol activity was not as much strong as compound H in stimulating expression of the *KL* promoter-containing reporter in the transfected kidney cells. It is likely that the endogenous inducer, calcitriol, may not be able to activate *KL* expression if a prerequisite condition exists, i.e., DNA methylation. Therefore, we expect that *KL* expression could not be induced by a transcription factor alone under the methylation condition. Increased binding of Pax4 and Kid3 to the *KL* promoter by compound H treatment support our notion; DNA demethylation might be a favorable condition for the binding of these TFs (Figure 4B).

DNA methylation is a general mechanism that blocks a gene expression. Methylated DNA is hardly occupied by transcription factors, so that demethylation is required for proper binding of the TFs, such as Pax4 and Kid3, to the promoter. Indeed, we found that compound H treatment increased demethylation and enhanced binding of Pax4 and Kid3 to the *Kl* promoter (Figure 4). Downregulation of Kid3 potentiated compound H-induced sKl expression, whereas downregulation of Pax4 attenuated the activity of compound H (Figure 5). Although silencing of Kid3 potentiated the effect of compound H (Figure 5D-F), it may be too early to conclude that Kid3 is a repressor for Kl expression. Kid3 is a C2H2 (Krüppel-like) zinc finger protein which is involved in the development [28]. We speculate that Kid3 might be an integrator which resists against any signals that could change the integrity in the cells. We found that cells were detached from the culture dish surface and died when Kid3 expression was decreased.

An increase in DNA methylation on the CpG islands of *Kl* gene might be an important contributor for the age-related decrease in KL expression [14]. In the artificial absence of methylation by compound H, the transcription factors could bind to the promoter and activate gene expression. We found that the compound H-induced demethylation was accomplished through an active process by stimulating demethylases. Inhibition of methylation by a methylase inhibitor, 5-azacytidine, failed to induce Kl expression. As we expected, DNA demethylase activity was increased in the cells treated with compound H only for 6hr which is too short for methylase inhibitors to exert their activity. Enhanced demethylase activity by compound H also resulted in demethylation on p16 gene promoter. Thus, compound H-mediated DNA demethylation may not be gene-specific. Selective induction of *KL* expression should be considered when screening for an anti-aging therapeutic agent. We showed in a recent study that compound H also induced klotho expression, increased circulating klotho levels, and improved arterial stiffening in aged mice (unpublished data). Since compound H has not been tested for its safety, caution is needed for inducing KL *in vivo* because it may cause unwanted effects.

This is an initial study to explore whether and how compound H may induce kotho expression. The finding showed that the compound H activated DNA demethylase leading to demethylation and klotho expression. However, the mechanism of compound H-induced activation of DNA demethylase is not clear. A further study is warranted to investigate how compound H acts on demethylase.

## MATERIALS AND METHODS

### Plasmid constructions

The pHKP-luc reporter plasmid was kindly provided by Dr. Kadir Turan [21]. This plasmid contains -2.0 kb upstream sequence from the translation start codon of human *KL* gene. Reporter plasmids containing a fragment of the human KL promoter were constructed by cutting the pHKP-luc plasmid with restriction enzymes and followed by ligation with klenow fragment. The restriction enzymes used for the constructions are listed in Figure 2C. Each reporter plasmid containing the fragment was named following the restriction enzyme used for cutting with Not1. DNA sequences of every plasmid used for the experiments were confirmed by DNA sequencing. siRNA for Kid3 and Pax4 were purchased from Santa Cruz Biotechnology.

### Transient co-transfection assay

Mammalian cells (HRCE, HK-2, HEK293 and DCT) were used for the transfection assay. HRCE (human renal cortical epithelial) cells were purchased from Lonza. HK-2 (human kidney proximal tubule) cells and HEK293 (human embryonic kidney) cells were purchased from ATCC. A DCT (mouse distal convoluted tubule) cell line was kindly provided by Peter Friedman in University of Pittsburgh [29]. X-treme GENE transfection reagent from Roche Applied Science was used for transfection. For analyses of *KL* gene expression, plasmid reporters in which fragments of human *KL* gene promoter drive the fire fly luciferase gene expression were used. For transfection control, a reporter plasmid expressing renilla luciferase under control of thymidine kinase promoter was used. Luciferase assays were conducted using Dual-Luciferase^®^ Reporter Assay System from Promega. Fire fly luciferase activity was divided by matched renilla luciferase activity to present relative luciferase activity. Transfected cells were treated with DMSO or small molecules for the indicated time period in each result after 24 h of transfection.

### Reverse-transcription polymerase chain reaction (RT-PCR)

Semi-quantitative RT-PCR was conducted with purified RNA from mammalian cells [30, 31]. One μg of RNAs purified using RNeasy kit (Qiagen) was used for synthesis of the first strand cDNA with a random hexamer (Roche) and Superscript III first-strand synthesis kit (Invitrogen). PCR was conducted with high fidelity 2X PCR master mix (NEB).

### DNA pull-down experiments

All of the processes were done following the protocol published by BL Jutras et al. with minor modifications [32]. Biotin-labeled 200-bases DNA encompassing human *KL* promoter/enhancer was hybridized with a 200-bases complementary DNA, with which streptavidin-coated Dynabeads from Life Technologies (Grand Island, NY) were labeled. Nuclear extracts prepared from DCT cells treated with DMSO or compound H for 24 h were incubated with the labeled magnetic beads at room temperature for 30 min. Binding proteins to the DNA were purified with a DynaMag™ (Life Technologies) and analyzed on SDS-PAGE followed by western blot.

### DNA methylation analysis

Methylation on the CpG islands of the mouse *Kl* gene was analyzed with a kit ‘Promoter methylation PCR kit’ from the Affymetrix (Santa Clara, CA) by following the manufacturer's protocol. Briefly, mouse DCT cells treated with DMSO, the compound H, or 5-azacytidin (a methyltransferase inhibitor) were collected to purifying genomic DNAs, which were cut with Mse 1 restriction enzyme to make fragments of the DNAs. For quantification of DNA methylation, the fragmented DNAs were incubated with Mecp2, a protein exclusively binds to methylated DNA, and then the Mecp2-bound DNAs were captured to the beads pre-labeled with an anti-Mecp2 antibody in a column. After heavy washings to remove unmethylated DNAs, methylated DNAs were eluted and used for amplifying CpG islands of *KL* gene with PCR. An increase of the amplified PCR products indicates an increase of methylation and vice versa.

### DNA demethylase assay

The DNA demethylase assay kit was purchased from Epigentek. The process was conducted by following manufacturer's protocol. Briefly, pre-methylated DNA substrates were incubated with nuclear extracts purified from the DCT cells treated with DMSO or compound H (5 M) for 24 hr. DNA demethylase activity was quantified by measuring the amounts of left over methylated DNA using a specific antibody for methylated cytosine. Low observance indicates there is a strong DNA demethylase activity.

### Western blot

The western blot analysis was performed as described in our recent studies [33–37]. Ten μg protein from whole cell extracts were loaded onto each well of Criterion™ TGX^™^ precast gel from Bio-Rad. Protein amounts were quantified using the BCA protein assay kit from Thermo Scientific following manufacturer's protocol. Migrated protein bands in SDS-PAGE were transferred onto nitrocellulose membrane (0.2 μm) from Bio-Rad using a Bio-Rad trans-blot turbo system. Expression levels of KL proteins were measured with an anti-mouse KL antibody from R & D systems or an anti-human KL antibody from Cosmo bio (Clone No. KM2076). An anti-actin antibody was purchased from Abcam. Fluorescence-labeled secondary antibodies were purchased from Invitrogen. Protein bands were developed and analyzed with the ChemiDoc™ MP imaging system from Bio-Rad.

### Statistical analysis

The data were analyzed by one-way analysis of variance (ANOVA). The unpaired t-test was used for comparisons between two groups. Significance was set at a 95% confidence limit.

## SUPPLEMENTARY MATERIALS FIGURES


