# Supplementary material for: Induction of anti-aging gene klotho with a small chemical compound that demethylates CpG islands
Source: Oncotarget. 2017 Jun 22;8(29):46745–55. doi: 10.18632/oncotarget.18608 (PMC5564520; doi:10.18632/oncotarget.18608)
Supplement: Supplementary file 1 [file oncotarget-08-46745-s001.pdf]

Induction of anti-aging gene klotho with a small chemical compound that demethylates CpG Islands

Supplementary Material

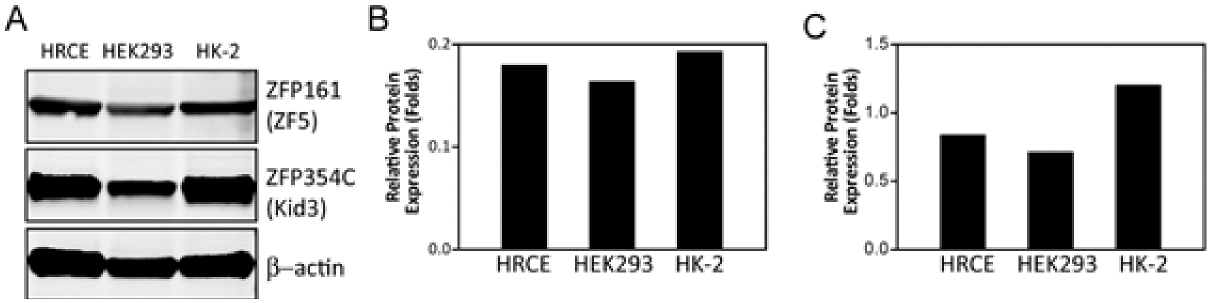

Supplementary Figure 1: Protein expression of ZF5 and Kid3. Whole lysates were prepared from the cells as described in the method section. Sixty µg of the lysate was loaded onto the wells and separated on SDS-PAGE. Expression level of the proteins was detected via western blot and quantified by the image lab software in theChemiDoc™ MP imaging system. (**A**) Western blot detection of the ZF5 and Kid3. (**B**) Quantification of ZF5 expression after normalization with β-actin expression level. (C) Quantification of Kid3 expression after normalization with β-actin expression level.

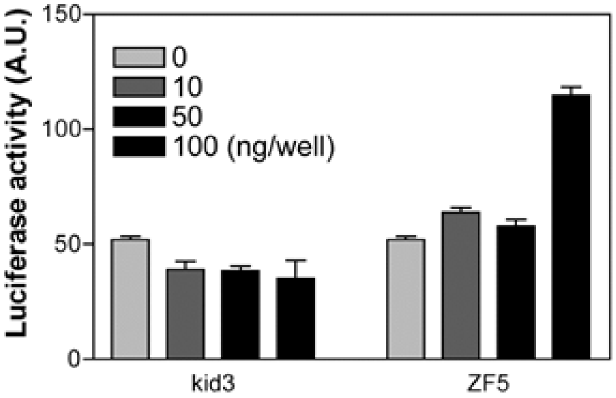

Supplementary Figure 2: Marginal repression and activation of the reporter expression by overexpression of kid3 or ZF5, respectively. The HRCE cells were transfected with the pHKP-luc reporter plasmid and an expression plasmid encoding Kid3 or ZF5. Expressed luciferase gene was quantified using Dual-Luciferase™ Reporter Assay System.
